# Editorial: Reviews in gastroenterology 2023

**DOI:** 10.3389/fmed.2025.1721878

**Published:** 2025-11-10

**Authors:** Junjie Zhang, Huan Tong

**Affiliations:** 1 West China School of Medicine, Sichuan University, Chengdu, China; 2 Department of Gastroenterology and Hepatology, West China Hospital, Chengdu, China

**Keywords:** acute upper gastrointestinal bleeding, electrogastrogram, functional constipation, rectal cancer, gastroenterology

The Research Topic of *Reviews in Gastroenterology 2023* focuses on cutting-edge knowledge of gastroenterology, and four reviews focus on acute critical risk stratification, noninvasive diagnosis of functional diseases, safe treatment for elderly patients, and precise positioning of malignant tumors. The four included papers reveal the frontiers of gastroenterology and provide new insight into gastroenterology.

Acute upper gastrointestinal bleeding (AUGIB) is a common emergency in clinical practice, raising many concerns from practitioners. Although progress has been made in the past decade, the potential fatality of AIGIB still requires more in-depth studies. As machine learning (ML) has emerged to assist and change medical daily work, 73 papers published from 2013 to 2023 on ML in AIGIB have been identified from the Web of Science database. Li et al. summarized these studies and reported that ML has uneven advantages in analyzing multidimensional medical data to predict patient prognosis. Among these ML models, a gradient-boosted ML model significantly outperforms Rockall, AIMS65, and Glasgow-Blatchford scores in prognosis prediction. However, most studies do not categorize datasets into nonvariceal and variceal bleeding datasets, which might compromise the accuracy of the model, as the treatment strategy differs across these subgroups. In the future, it is necessary to establish subtype-specific databases and promote multicenter data sharing to improve the interpretability of ML models.

Unlike the wide application of electrocardiograms in cardiology, electrogastrograms (ESGs) are less widely used in gastroenterology, although they were first used in the early 1920s. The poor popularity of EGG lies in its unstable results and the lack of a unified standard. Oczka et al. not only provided information on the electrical activity of the stomach but also listed the details of measurement methodology, methods of filtration and analysis, and comparative analysis of EGG procedures. This knowledge is helpful for overcoming the shortcomings of EGG, and EGG might hopefully be expected to become a common diagnostic tool for functional gastrointestinal diseases and gastrointestinal motility disorders if confounding factors of EGG are well controlled.

With respect to the treatment of functional gastrointestinal diseases, our topic included one systemic review on functional constipation. Among seniors aged older than 60 years, the prevalence of constipation reaches 22%, making functional constipation a major issue. Long-term illness is prone to induce cardiovascular and cerebrovascular accidents. A meta-analysis (Song et al.) focused on the efficacy of acupuncture in treating functional constipation. It included eight randomized controlled trials and favored acupuncture in elderly patients with functional constipation, with a better effective response and improvement in complete spontaneous bowel movements. However, the limitations of the evidence, such as the small sample size of each study, the unclear randomization, the use of a subjective acupuncture procedure, and the lack of long-term follow-up, alerted physicians to the efficacy of acupuncture on functional constipation in seniors.

For the third leading cause of fatal malignant tumors worldwide, rectal cancer, MRI is optimal for its diagnosis, but its limited expertise could hinder its diagnostic accuracy. One review (Yang et al.) described different algorithms, including convolutional neural networks, recurrent neural networks, and graph neural networks, and suggested that deep learning (DL) models based on the U-Net series algorithm achieve pixel-level segmentation of MRI lesions through encoder-decoder symmetrical structures and skip connections with Dice coefficients close to those of radiologists. However, most studies are based on 2D segmentation, whereas 3D spatial view is needed in the clinic. The datasets were mostly small samples from a single center and focused on T2W MR images, with insufficient exploration of T1W MR images. Thus, machine learning models still need improvements in their application in the diagnosis of rectal cancer.

The four reviews highlight new understandings and progress in certain sections of gastroenterology. These sections are unfinished with ongoing investigations and advances, which would benefit suffering patients. New knowledge is expected to emerge in the next Research Topic, *Reviews in Gastroenterology 2025*.

